# Extranodal NK/T-cell lymphoma, nasal type with extensive cardiopulmonary involvement

**DOI:** 10.4322/acr.2021.246

**Published:** 2021-03-19

**Authors:** Mario L. Marques-Piubelli, Gabriel Teixeira Montezuma Sales, Letícia Campos Clemente, Lidiane Inês Rosa, Mariana Lorenzi Savioli, Ricardo Pires Alvim, Raquel Megale Moreira, Fernando Pereira Frassetto, Ellen Caroline Toledo do Nascimento, Sheila Aparecida Coelho Siqueira

**Affiliations:** 1 Universidade de São Paulo, Faculdade de Medicina, Departamento de Patologia. São Paulo, SP, Brasil; 2 The University of Texas MD Anderson Cancer Center, Department of Translational Molecular Pathology. Houston, Texas, USA; 3 Universidade de São Paulo, Faculdade de Medicina, Departamento de Nefrologia. São Paulo, SP, Brasil; 4 Universidade de São Paulo, Faculdade de Medicina, Departamento de Hematologia e Hemoterapia. São Paulo, SP, Brasil; 5 Universidade de São Paulo, Faculdade de Medicina, Departamento de Neurologia. São Paulo, SP, Brasil

**Keywords:** Lymphoma, Extranodal NK-T-Cell, Epstein-Barr Virus Infections, Lymphoma, Lymphoma, Non-Hodgkin

## Abstract

Extranodal NK/T-cell lymphoma, nasal type (ENKTL-NT) is a rare type of Non-Hodgkin’s lymphoma, which usually presents with extranodal involvement and affects the nasal/upper aerodigestive tract in the classical presentation. Herein, we report the case of a 31-year-old, previously healthy, male patient diagnosed with ENKTL-NT with the involvement of the lung parenchyma and heart. Unfortunately, due to the rapid disease progression, the diagnosis was performed only at the autopsy. The authors highlight the rare clinical presentation of this type of lymphoma, as well as the challenging anatomopathological diagnosis in necrotic samples.

## INTRODUCTION

Extranodal NK/T-cell lymphoma, Nasal Type (ENKTL-NT) is an aggressive and rare type of T-Cell lymphoma associated with Epstein-Barr Virus (EBV) infection and derived from NK cells or cytotoxic T-cells.^1^ It was called “lethal midline granuloma” in the past due to the involvement of midline facial area. East Asians and Native Americans in Central and South America are the most commonly affected populations.^2^ We report a case of a 31-year old male with ENKTL-NT who had an atypical clinical presentation due the extensive cardiopulmonary involvement.

## CASE REPORT

A 31-year-old male was admitted to the emergency facility complaining of low back pain, daily fever, fatigue and hematuria over the last 10 days preceded by a weight loss of 8 kg for the last 2 months. He also presented epistaxis, hemoptysis, gross hematuria, dysphagia, dysphonia, and hearing loss over the last 24 hours. His medical history included recurrent episodes of sinusitis treated with antibiotics.

On admission, he was pale, dehydrated, pulse rate was 113 beats/min and blood pressure 93 x 52 mmHg. He also presented hypoxemic but improved after oxygen supplementation. Pulmonary examination showed diffuse rhonchi, while the cardiac and abdominal examination was normal. The initial neurological examination showed global weakness (muscle strength grade IV/V) and hypotonia in all four limbs and areflexia. The patient also presented with neurosensorial deafness (VIII cranial nerve) and partial palsy in cranial nerves III, IV, and VI. Peripheral facial weakness and dysphagia were also present, indicating involvement of VII cranial and bulbar nerves (IX, X, and XI), respectively.

The initial laboratory work-up showed mild anemia (Hemoglobin: 11.7 g/dL, Reference Value [RV]: 13.5 - 17.5 g/dL; Hematocrit: 31.2%, RV: 41 - 53%) and leukopenia (WBC: 3.140/mm^3^, RV: 4.500-11.000/mm^3^). Ferritin was 2800 ng/ml (RV: 20-250 ng/ml). Liver enzymes, lactate dehydrogenase (LDH), creatinine, urea, complement 3 (C3), complement 4 (C4), and creatine phosphokinase (CPK) were slightly increased. Serology for syphilis, hepatitis B and C, rheumatoid factor (RF), antinuclear antibody (ANA), and anti-neutrophil cytoplasmic autoantibody (ANCA) were negative. Urinalysis showed hematuria.

The thoracic computed tomography (CT) showed multiple nodules diffusely distributed in the lungs' parenchyma, with ground-glass opacity. The pleura and lymph nodes were not involved. The cranial CT showed thickening of the right ethmoid sinus wall.

A possible diagnosis of granulomatosis with Polyangiitis was made and therapy with piperacillin/tazobactam, vancomycin, and methylprednisolone was initiated. On the third day after admission, he underwent to a transbronchial biopsy, which revealed no significant changes of the respiratory epithelium and tested negative for yeast and acid-fast bacilli. Bronchoalveolar lavage culture showed *Acinetobacter baumanii complex*. A kidney biopsy was performed and showed mild acute tubular necrosis and hyaline arteriolosclerosis. On the sixth day of hospitalization, the patient developed progressive shortness of breath and septic shock. He was submitted to endotracheal intubation and Amphotericin B and Colistin were administrated. A sinus biopsy was also performed and showed intense necrosis with polymorphonuclear inflammatory infiltrate. Bone marrow aspirate showed hypocellularity and the biopsy was normocellular, both with normal maturation of the hematopoietic precursors. The flow cytometry immunophenotype was normal, and there was no clonal rearrangement of the T-cell receptor (TCR) genes. The cerebrospinal fluid (CSF) analysis showed 180 cells (49% lymphocytes, 44% neutrophils, 6% monocytes) with 83,200 red blood cells, a protein level of 271 mg/dl, and a glucose level of 127 mg/dl. The polymerase chain reaction (PCR) tests for cytomegalovirus, EBV, herpes simplex virus I/II, varicella-zoster virus and tuberculosis were negative, as well as the culture for aerobic and anaerobic bacterial. Concurrent flow cytometry of the CSF showed 61% NK lymphocytes, 36% mature T-cells and 5% polyclonal B-cells. The NK cells were sCD3 negative, CD16 positive, and CD56 positive.

Due to a non-definitive diagnosis and a suspicion of lymphoproliferative disorder, an open lung biopsy was performed on the 17^th^ day of hospitalization. However, hours after the procedure, the patient developed refractory hemodynamic instability, followed by death.

## AUTOPSY FINDINGS

On external examination, the corpse showed pale mucosae and a thoracic wound with no evidence of postoperative complications.

**Figure 1:**
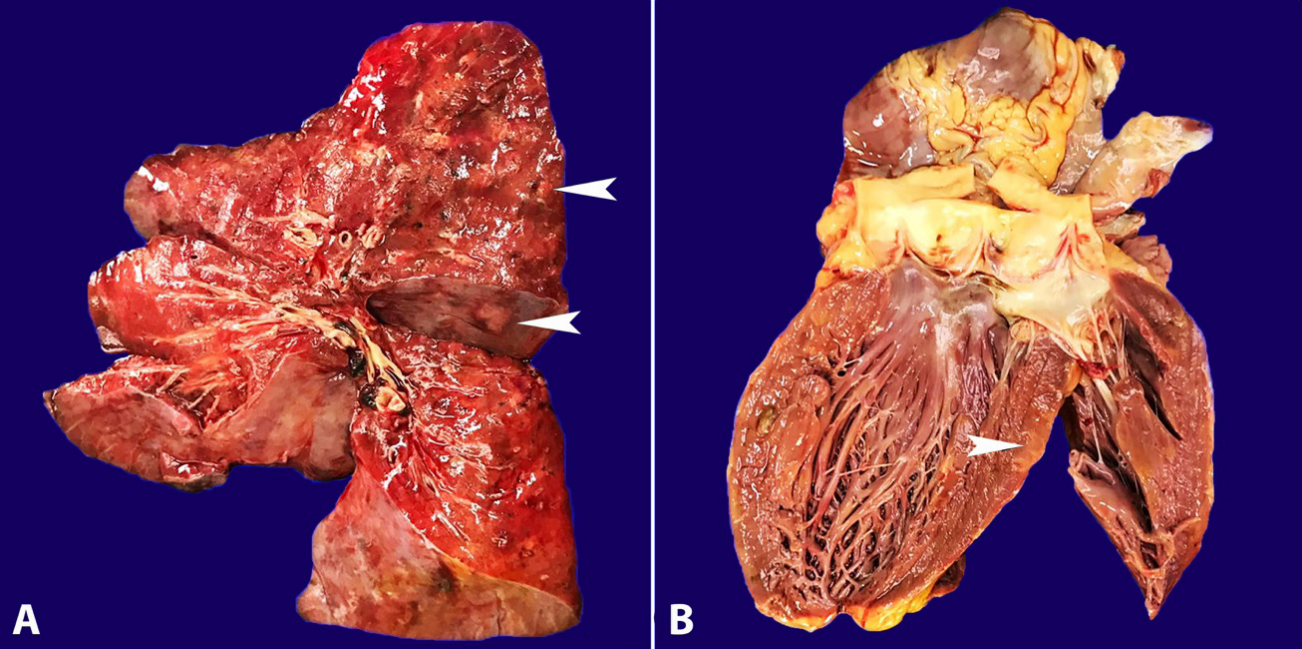
Macroscopic appearance of lung and heart. **A –** Gross view of the lung with multiple greyish nodular lesions (white arrows) in a background of a hemorrhagic parenchyma; **B –** Gross view of the heart shows very delicate irregular areas of pale and greyish color in the left ventricle (black arrow).

**Figure 2:**
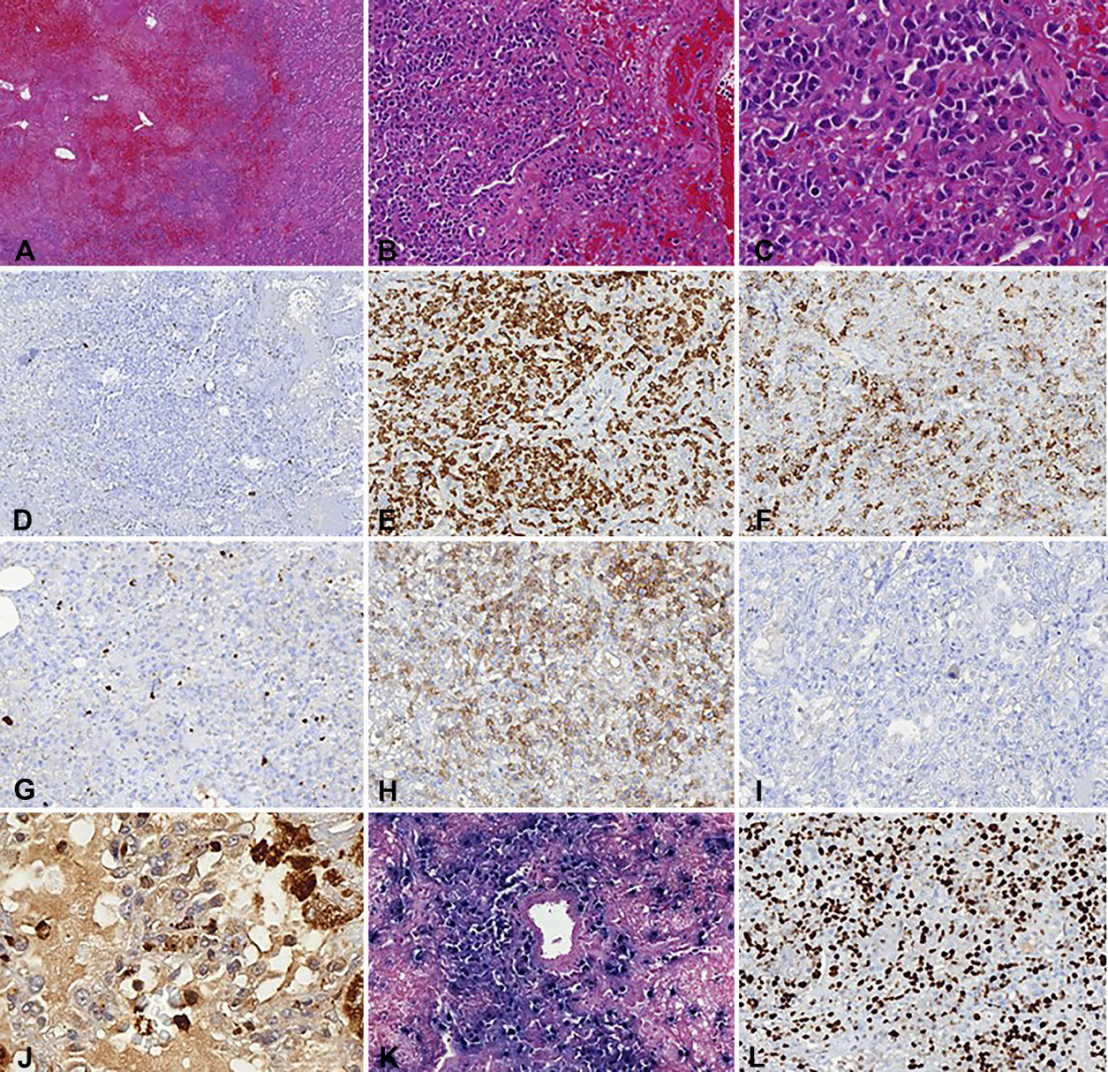
Histological appearance of a lung nodule: A - intense and diffuse hemorrhage, necrosis of the alveolar spaces, and diffuse lymphoid infiltrate (H&E, 40x); B - intense and atypical lymphoid infiltrate with angiocentric distribution (H&E, 100x); C - atypical lymphoid infiltrate composed by medium sized cells, irregular nuclei contour, condensed chromatin, and scant cytoplasm (H&E, 400x); D - immunohistochemistry for CD20 shows negative stain for lymphoma cells and positive reactive B-cells in the background (50x); E - immunohistochemistry for CD3 stain shows partial and strong positivity of the lymphoma cells (100x); F - immunohistochemistry for CD30 stain shows weak and partial positivity of the lymphoma cells (100x); G - immunohistochemistry for CD15 shows negativity of the lymphoma cells (100x); H - immunohistochemistry stain for CD56 shows diffuse and moderate stain of the lymphoma cells (100x); I - immunohistochemistry for ALK-1 protein shows negative stain of the lymphoma cells (100x); J - immunohistochemistry for CD68 shows negativity of the lymphoma cells and positivity of macrophages in the background (400x); K - *in situ* hybridization to EBV-encoded RNA (EBER) shows positivity in the lymphoma cells disposed in an angiocentric pattern (100x); L - Immunohistochemistry for Ki-67 shows high proliferation index (90%) in the lymphoma cells (100x).

There were large and bilateral pleural effusions of yellow citrine color. Both lungs showed multiple, round, fibroelastic, and whitish diffusely distributed nodules ranging from 0.3 cm to 2.0 cm (Figure 1A). The histological sections from the nodular areas showed the same findings as the open lung biopsy. There was an intense atypical lymphoid infiltrate composed of medium to large cells with irregular nuclei contours, hyperchromatic chromatin and no evident nucleoli. The upper airways had no significant histological changes and the adjacent lung parenchyma showed recent alveolar hemorrhage, diffuse alveolar damage, coagulative necrosis, pneumocytes hyperplasia, and microthrombi formation. The immunohistochemistry study was performed and showed positivity for εCD3 (Ventana, clone 2GV6), CD30 (Ventana, clone Ber-H2), and CD56 (Ventana, clone 123C3) in the atypical cells. The association with EBV was proven by in situ hybridization to EBV-encoded RNA (EBER). The Ki-67 (Ventana, clone 30-9) proliferation index in was 90%. The neoplastic cells were negative for CD20 (Ventana, clone L26), CD15 (Ventana, clone MMA), CD68 (Ventana, clone KP-1), and ALK-1 protein (Ventana, clone ALK01), supporting the diagnosis of Extranodal NK/T-cell lymphoma, nasal type (ENKTL-NT) (Figure 2).

**Figure 3:**
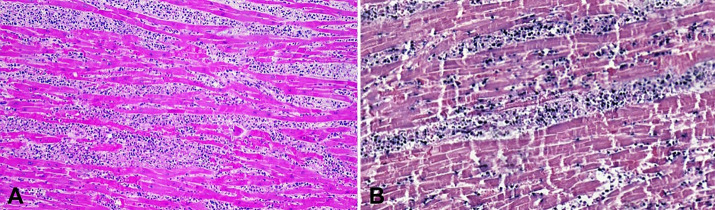
Photomicrographs of the heart: **A –** Hematoxylin & Eosin (H&E) section shows cardiomyocytes with intense and interstitial infiltrate composed by atypical lymphoid cells of small to medium size (100x); **B –** *in situ* hybridization to EBV-encoded RNA (EBER) showing diffuse positivity of the atypical lymphoid cells (200x).

The heart presented with normal valves and the myocardium showed pale greyish areas, which were more prominent in the left ventricle (Figure 1B). An intense interstitial infiltration of the cardiomyocytes by lymphoma cells was observed in these areas (Figure 3).

There was no evidence of lymphoma infiltration in the central nervous system (CNS), leptomeninges, and cranial nerves. The histological sections of the encephalon showed an increase of the perivascular space associated with perivascular hemorrhage, acute neuronal injury, and foci of oligodendroglial reactivity.

The liver and spleen were grossly congested. Upon histology, the liver showed reactive findings related to sepsis, such as mild portal polyclonal lymphoplasmacytic infiltrate. Similarly, the spleen presented with diffuse congestion and multiple necrotic areas, with no evidence of lymphoma infiltration.

The right kidney sections showed foci of hemorrhage in corticomedullary junction, early microscopic infarction of the cortex, and acute tubular necrosis. The bone marrow analysis from right iliac crest showed normocellular marrow (70% of cellularity) with trilineage cell maturation and no evidence of lymphoma infiltration.

According to the autopsy findings, we interpreted the immediate cause of death as a diffuse alveolar damage secondary to extensive lymphoma infiltration of the lung and the heart.

## DISCUSSION

The case reported herein describes a young adult man who died due to a cardiopulmonary involvement by ENKTL-NT diagnosed at autopsy. This aggressive type of lymphoma is very rare and corresponds to 0.2% of all Non-Hodgkin’s lymphomas and 1-2% of all NK/T-cell lymphomas with higher incidence in middle-aged males.^1^ ^,^ ^2^

Approximately 80% of ENKTL-NT cases occur in the upper aerodigestive tract, such as the nasopharynx, paranasal sinuses, and orbit.^3^ Thus, most patients have a clinical presentation of upper airway obstruction, purulent discharge, epistaxis, and facial mass.^4^ Patients with involvement of these areas, regardless of the dissemination to other sites, are considered as a nasal presentation. However, the exclusive involvement of extranasal sites may occur in 20% of cases. It can present with varied clinical features, making the diagnosis extremely challenging.^3^ ^,^ ^5^ ^,^ ^6^ The main affected sites include the digestive tract, skin, adrenal glands, eyes, breast, penis, CNS, and testis.^1^ ^,^ ^4^ ^,^ ^7^ ^-^ ^9^ The bone marrow involvement is rare and seen in less than 20% of the patients, making diagnosis even more difficult.^10^

The involvement of the heart by any type of lymphoma is infrequent, corresponding to 10% to 25% of all autopsied lymphoma cases.^11^ It is more common in T-cell lymphomas, and this hidden diagnosis is usually made at autopsies.^11^ ^,^ ^12^ Cardiopulmonary involvement by ENKTL-NT is also extremely rare and reported in the literature only as isolated case reports.^8^ ^,^ ^13^ ^-^ ^17^ In these cases, the heart involvement has been described as pericardial effusion, contiguous lesions or interstitial myocardial infiltration. The clinical manifestations of these cases were broad and associated with myocardial ischemia, valvar disease, outflow obstruction, cardiac tamponade, heart failure and death.

The CNS involvement is rare and occurs in less than 10% of all cases of ENKTL-NT.^18^ Due to varied neurological presentations, the CNS imaging evaluation is mandatory, and the diagnosis of paraneoplastic neurological syndromes should be performed as an exclusion diagnosis.^17^ The CNS involvement can be determined clinically or pathologically by cerebrospinal fluid analysis by cytology/flow cytometry, clonality or histologic diagnosis on brain biopsy.^18^ Importantly, the involvement of CNS in these cases is usually associated with more than one extranodal site and the prognosis is extremely poor.^18^ In the presented case, we attributed the neurological symptoms to a paraneoplastic syndrome. Although there was evidence of NK-cell expansion in the CSF, it was not possible to confirm clonality due the small number of cells in the sample. Importantly, there was no histological or immunophenotypically evidence of lymphoma infiltration of the encephalon, cranial nerves, leptomeninges, pons or medulla oblongata in the autopsy findings.

The causes of death in ENKTL-NT were recently explored in a recent study performed by Mei *et al*.^19^ In this large retrospective cohort study, the authors included 163 patients. The cause of death was related to ENKTL-NT in 52.76% of the cases followed by other malignant neoplasia, infectious and heart diseases. The diagnosis at an older age and more advanced disease stage were related to increased mortality.

The histopathologic diagnosis of NK/T-cells malignancies, especially in ENKTL-NT, is challenging.^1^ ^,^ ^9^ As occurred in our case, the diagnosis was delayed due to limited biopsies and the presence of extensive necrosis. Thus, the histopathological confirmation of NK/T-cell clonality is frequently impaired.^1^ The morphologic presentation in classical cases consists of a neoplastic infiltration with markedly atypia, pleomorphism and wide size variation. The common presence of a mixed inflammatory Infiltrate in the background may mask the diagnosis.^1^ ^,^ ^2^ The angiocentric distribution of the neoplastic cells, leading to vessel obstruction and necrosis, is also a consistent morphological finding.^1^ ^,^ ^7^ The NK-cells are the classical cells of origin; however, a T-cell phenotype has also been described in a minority of cases, which makes the diagnosis even more challenging.^1^ ^-^ ^3^ ^,^ ^5^ ^,^ ^6^ EBV has a fundamental role in the pathogenesis of ETNC-NT and it is present in almost all cases with a type 2 latency.^1^ ^,^ ^2^ ^,^ ^5^ ^,^ ^6^ In this latency type, the virus restricts its gene expression and is positive only for EBNA-1, LMP-1, LMP-2, and EBER.^20^ The detection by *in situ* hybridization is more sensitive than LMP-1 in paraffin-embedded tissue.^2^

As EBV is virtually present in all cases, testing is mandatory in all suspected cases.^2^ ^,^ ^6^ As considered during the clinical investigation of our case, the most important differential diagnoses are granulomatosis with polyangiitis, fungal infection (such as Aspergillosis), lymphomatoid granulomatosis, and peripheral T-cell lymphoma, not otherwise specified (PTCL, NOS).^1^ ^,^ ^2^

## CONCLUSION

We interpreted this case as a rare and atypical clinical presentation of ENKTL-NT and highlighted the difficulties in performing a precise diagnosis in the setting of a dramatic and broad clinical course. Medical staff should keep in mind the investigation of this disease in the context of acute clinical presentation of heterogeneous symptoms and extranodal lesions.

## References

[1] Haverkos BM, Pan Z, Gru AA (2016). Extranodal NK/T Cell Lymphoma, Nasal Type (ENKTL-NT): an update on epidemiology, clinical presentation, and natural history in north american and european cases. Curr Hematol Malig Rep.

[2] Kim WY, Montes-Mojarro IA, Fend F, Quintanilla-Martinez L (2019). Epstein-barr virus-associated T and NK-Cell Lymphoproliferative Diseases. Front Pediatr.

[3] Au WY, Weisenburger DD, Intragumtornchai T (2009). Clinical differences between nasal and extranasal natural killer/T-cell lymphoma: a study of 136 cases from the International Peripheral T-Cell Lymphoma Project. Blood.

[4] Kim TM, Lee SY, Jeon YK (2008). Clinical heterogeneity of extranodal NK/T-cell lymphoma, nasal type: a national survey of the Korean Cancer Study Group. Ann Oncol.

[5] Li S, Feng X, Li T (2013). Extranodal NK/T-cell lymphoma, nasal type: a report of 73 cases at MD Anderson Cancer Center. Am J Surg Pathol.

[6] Gualco G, Domeny-Duarte P, Chioato L, Barber G, Natkunam Y, Bacchi CE (2011). Clinicopathologic and molecular features of 122 Brazilian cases of nodal and extranodal NK/T-cell lymphoma, nasal type, with EBV subtyping analysis. Am J Surg Pathol.

[7] Dojcinov SD, Fend F, Quintanilla-Martinez L (2018). EBV-Positive Lymphoproliferations of B- T- and NK-Cell Derivation in Non-Immunocompromised Hosts. Pathogens.

[8] Lepeak LM, Yang DT, Chang JE (2011). Extranodal NK/T-cell lymphoma presenting with primary cardiac involvement. Hematol Rep.

[9] Wang X, Gong Z, Li SX, Yan W, Song Y (2017). Extranodal nasal-type natural killer/T-cell lymphoma with penile involvement: a case report and review of the literature. BMC Urol.

[10] Li S, Feng X, Li T (2013). Extranodal NK/T-cell Lymphoma, Nasal Type: A report of 73 Cases at MD Anderson Cancer Center. Am J Surg Pathol.

[11] Chinen K, Izumo T (2005). Cardiac involvement by malignant lymphoma: a clinicopathologic study of 25 autopsy cases based on the WHO classification. Ann Hematol.

[12] McDonnell PJ, Mann RB, Bulkley BH (1982). Involvement of the heart by malignant lymphoma: A clinicopathologic study. Cancer.

[13] Kawasaki H, Shigeno K, Ohnishi K (2008). A case of primary cutaneous natural killer/T-cell lymphoma, nasal type, directly invading to the heart. Leuk Lymphoma.

[14] Baek YS, Shin SH, Yi HG (2014). Cardiac involvement in CD56 negative primary pancreatic extranodal NK/T-cell lymphoma, nasal type, presenting with ventricular tachycardia during the early stages of chemotherapy. Intern Med.

[15] Kanesvaran R, Tao M, Huat IT, Weng DT, Eng DN, Thye LS (2009). Malignant arrhythmia: a case report of nasal NK/T-cell lymphoma with cardiac involvement. Acta Oncol.

[16] Li Y, Damjanov I (2016). Extranodal NK/T Cell Lymphoma Causing Cardiorespiratory Failure. Case Rep Hematol.

[17] Deepti AN, Noone ML, Mahadevan A (2008). Primary cardiac cytotoxic T-cell lymphoma presenting with neurological deficits: a case report. Cardiovasc Pathol.

[18] Gurion R, Mehta N, Migliacci JC (2016). Central nervous system involvement in T-cell lymphoma: A single center experience. Acta Oncol.

[19] Mei M, Wang Y, Zhang M (2019). Causes of mortality in cases with extra nodal natural killer/T-cell lymphoma, nasal type: A cohort study. PLoS One.

[20] Marques-Piubelli ML, Salas YI, Pachas C, Becker-Hecker R, Vega F, Miranda RN (2020). Epstein-Barr virus-associated B-cell lymphoproliferative disorders and lymphomas: a review. Pathology.

